# Record Linkage for Malaria Deaths Data Recovery and Surveillance in Brazil

**DOI:** 10.3390/tropicalmed8120519

**Published:** 2023-12-14

**Authors:** Klauss Kleydmann Sabino Garcia, Danielly Batista Xavier, Seyi Soremekun, Amanda Amaral Abrahão, Chris Drakeley, Walter Massa Ramalho, André M. Siqueira

**Affiliations:** 1 Center of Tropical Medicine, University of Brasilia, Brasília 70904-970, Brazil; 2 Escola Superior de Agricultura Luís de Queiroz, Universidade de São Paulo, Piracicaba 13418-900, Brazil; 3 Department of Infection Biology, Faculty of Infectious and Tropical Diseases, London School of Hygiene & Tropical Medicine, University of London, London WC1E 7HT, UK; 4 Faculty of Health Sciences, University of Brasilia, Brasilia 70910-900, Brazil; 5 Oswaldo Cruz Foundation, Evandro Chagas National Institute of Infectology, Rio de Janeiro 21040-360, Brazil

**Keywords:** malaria, epidemiology, public health, control, interrupted time series, Brazil

## Abstract

Objective: The objective is to describe the results and the methodological processes of record linkage for matching deaths and malaria cases. Methods: A descriptive cross-sectional study was conducted with probabilistic record linkage of death and malaria cases data in Brazil from 2011 to 2020 using death records from the Mortality Information System (SIM) and epidemiological data from the Notifiable Diseases Information System (Sinan) and Epidemiological Surveillance Information Systems for malaria (Sivep-Malaria). Three matching keys were used: patient’s name, date of birth, and mother’s name, with an analysis of cosine and Levenshtein dissimilarity measures. Results: A total of 490 malaria deaths were recorded in Brazil between 2011 and 2020. The record linkage resulted in the pairing of 216 deaths (44.0%). Pairings where all three matching keys were identical accounted for 30.1% of the total matched deaths, 39.4% of the matched deaths had two identical variables, and 30.5% had only one of the three key variables identical. The distribution of the variables of the matched deaths (216) was similar to the distribution of all recorded deaths (490). Out of the 216 matched deaths, 80 (37.0%) had poorly specified causes of death in the SIM. Conclusions: The record linkage allowed for the detailing of the data with additional information from other epidemiological systems. Record linkage enables data linkage between information systems that lack interoperability and is an extremely useful tool for refining health situation analyses and improving malaria death surveillance in Brazil.

## 1. Introduction

Brazil’s health surveillance system boasts a rich repository of epidemiological data stored in various Health Information Systems (HISs). Some of the HISs most frequently utilised by Health Surveillance include the Mortality Information System (SIM), housing information sourced from death certificates (DC); the Notifiable Diseases Information System (Sinan) and the Epidemiological Surveillance Information Systems (Sivep-Malária), both containing data from notification forms for diseases and notifiable conditions; the Hospital Information System (SIH), featuring data on hospital admissions; and the Live Birth Information System (SINASC), containing information on live births in Brazil [1].

The interoperability of these systems has not yet been achieved [1], and there is no single unique patient (user) identifier allowing the linkage between different databases. Historically, most records lack national individual identification numbers such as the Individual Taxpayer Registration (Cadastro de Pessoa Física—CPF) [2]. Consequently, when a need to correlate information across different databases arises, it becomes necessary to link data from the previously nominated HIS using probabilistic data linkage techniques.

Probabilistic linkage can be understood as a technique that considers a set of combination rules that analyse the probability of distinct records belonging to the same individual by calculating measures of similarity between open-field, categorical, date, or dissimilarity measures between numerical variables [3]. These techniques employ measures of similarity between records using variables such as name, mother’s name, date of birth, gender, and place of residence, among others [4].

Probabilistic data linkage has yielded favourable results in research across various fields of knowledge and provided evidence for quality health outcomes within the public health sector [5,6,7]. However, the linkage not only depends on specialised human resources but also relies on the quality of data within these HIS records, and the availability of computational processing power for the task [2].

Given the necessity to integrate information from different HISs and facing limitations related to data quality and accessibility, probabilistic data linkage methods (record linkage) have proven to be a viable alternative for this integration [3]. This method has shown versatility by working with a set of rules that allow the analysis of similarity between records. The technique also enables the assessment of possible related pairs as true or false and maximises the number of true pairs identified [8]. Therefore, probabilistic linkage techniques present better results than deterministic record linkage because its sensitivity allows one to find more true pairs than the deterministic one, which matches only perfect records between databases [2,9].

The HISs used in the epidemiological surveillance of malaria are Sivep-Malaria and Sinan. Malaria cases reported in the Amazon region (an endemic area that covers the northern region of Brazil and the states Maranhão and Mato Grosso) are notified in Sivep-Malaria, while cases outside the Amazon region (area not endemic) are reported in Sinan. These individual notification forms do not collect information about case progression or mortality [10,11]. The Amazon region is responsible for approximately 99% of malaria cases in Brazil, while the other 1% that happens in the non-endemic area is majorly imported from the Amazon region or other countries. Because the occurrence of malaria in the non-endemic region is uncommon, diagnosis and treatment may occur late [12]. Possibly due to these factors that are related to surveillance and assistance matters, the non-endemic region can present fatality rates varying between 0.8% and 3.3% [13]. In 2019, the fatality rate in the non-endemic region was 123 times greater than the lethality rate in the Amazon region, which was under 0.02% [14].

For the official monitoring of malaria-related deaths, the National Malaria Control and Prevention Program (NMCP) utilises SIM data, which encompasses deaths caused by different malaria species. To investigate specific issues related to malaria death surveillance, it is necessary to analyse information from different HISs together. It is crucial to have clarity regarding what is related to malaria-related deaths, especially considering that this is a preventable health event [15]. This information can promote actions that might prevent these deaths.

Therefore, in light of the need to enhance malaria death surveillance activities, this study considered it important to operationalise the linkage, while reporting the methodological processes, and describing the information retrieved from different HISs. This will facilitate the discussion of the procedures conducted, challenges encountered, and actions that can be developed with the newly available data.

The aim of this study is to describe the results and methodological processes used in data linkage (record linkage) for malaria-related deaths and cases of the disease. It is expected that this material can serve as a model for other record linkage processes and help malaria death surveillance.

## 2. Materials and Methods

It is a descriptive cross-sectional study that reports the methodological processes of record linkage technique for the validation of the technique for retrieving sociodemographic and epidemiological data on malaria-related deaths. Procedures and results of the data linkage method are described in this work. The data pertain to the total number of malaria-related deaths recorded in the SIM (ICD-10: B50, B51, B52, B53, and B54) and the total number of malaria-positive cases reported in Brazil in Sivep-Malaria and Sinan between 2011 and 2020.

From the SIM database, the following variables were utilised: date of death, date of birth, patient’s name, mother’s name, gender, age, race/ethnicity, education level, underlying cause of death, associated causes of death, place of death occurrence, medical care, death investigation, and occupation.

From the Sivep-Malaria and Sinan databases, the following variables were used: date of symptom onset, date of notification, date of birth, patient’s name, mother’s name, gender, age, race/ethnicity, education level, laboratory test results, and type of slide (new case slide or possible recurrence).

Descriptive statistics techniques were employed for data description. Confidence intervals for the relative frequencies of the studied variables were calculated to assess similarity between the distributions of paired data through record linkage compared to the original data. The chi-square test was applied to verify differences in variable distribution between the original records and the linked data. A significance level of 0.05 was adopted. When the *p*-value is greater than 0.05, it indicates no difference between the proportions of variables among the databases. In other words, the linked records in this scenario will exhibit a variable distribution similar to the distribution of the original database. The HIS records were also compared to identify possible inconsistencies between sociodemographic variables and *Plasmodium* species information. Additionally, the quantity of information marked as “unknown”, “blank”, or ambiguously defined as “unspecified malaria” was examined.

This work received favourable opinion number 51246121.0.0000.5558 from the Ethics Committee on Research of the Faculty of Medicine of the University of Brasília, and data access was obtained through an official request submitted to the Office of the Secretary of Health Surveillance and Environment of the Brazilian Ministry of Health (BMoH). Authorization to handle this data was granted by the Coordination-General of Information and Epidemiological Analysis (CGIAE) and the Coordination-General of Zoonoses and Vector-Borne Diseases Surveillance (CGZV). Data handling was conducted by CGIAE in a secure and monitored environment, following security protocols to prevent misuse or data leakage.

For the development of the record linkage R language and R Studio interface (version 4.2.1) were used [16]. The R script is available in Supplementary Material SI.

### Specific Linkage Procedures

Pre-planning: Variables of interest from the SIM, Sivep-Malaria, and Sinan databases were defined. For pairing, “Patient’s Name,” “Date of Birth,” and “Mother’s Name” were selected as linkage key variables, following suggestions from previous studies [2,17,18].

Data treatment: To perform data treatment and linkages, the tidyverse, lubridate, stringr, stringi, fuzzyjoin, stringdist, abjutils, and Rcpp packages from R software (version 4.2.1) were used. Data preparation followed the data cleaning and treatment methods previously reported [2]. To reduce errors and inconsistencies, an initial standardisation of key variables was performed in all three databases. All characters in the “Patient’s Name” and “Mother’s Name” variables were converted to uppercase. Name connectors such as “E,” “A,” “DA,” “DE,” “DO,” “DAS,” “DES,” and “DOS,” accents, symbols, and spaces were removed. Texts resembling “observations” were also removed from the “Patient’s Name” field, including numbers, punctuation marks, “KG” (kilogram), “PESO” (weight), “TEL” or “TL” or “FONE” (telephone), “OBS” (observation), and the words “unknown” or “unspecified.” The “Patient’s Name” variable was split into two new variables: “first name” and “last name” to increase the sensitivity of the pairing method.

All described procedures were applied to the SIM, Sivep-Malaria, and Sinan databases. Due to the high number of notifications in the Sivep-Malaria database, notifications from this system were divided into biennial blocks (2011–2012, 2012–2013... 2018–2019, 2019–2020) to facilitate data processing by the software and capture epidemiological information prior to death. Considering that malaria illness and death are acute events [19], there is no perceived loss of pairs in this blocking process.

Parameters and rules for linkage: probabilistic linkage was performed using the “fuzzyjoin” package, which requires defining parameters for comparing information from different databases to operate effectively. In this package, distance measures need to be defined for nominal, numeric, and date variables. Two distance measures used in the pairings are Levenshtein [20] for names and cosine [21] for last names. For dates, these same distance techniques can be chosen, considering possible incorrect entries.

In statistics, a measure of distance refers to a quantitative calculation used to determine the similarity or dissimilarity between data points, vectors, or distributions. Levenshtein distance measures the minimum number of single-character edits required to change one string into the other and the cosine distance measures the cosine of the angle between two vectors (in this case: names) [22].

The linkage considered the following keys simultaneously: “first name,” “last name,” “date of birth,” and “mother’s name.” Levenshtein distance value used was 3 for the “first name” and “date of birth,” while cosine distance was set at 0.5 and used for “last names” and “mother’s name.”

The value of 3 for Levenshtein distance allows up to 3 characters between records to be different and still to be considered a match, for example, “Danieli” and “Dannielly” and “1994-04-25” and “2094-04-26” (errors underlined) are possible to match. The use of value 2 in Levenshtein distance would make the parameter less sensitive. The Levenshtein distance value 3 (higher sensitivity than value 2) was used considering the common large amount of misspellings in first names and birth dates. The cosine distance can be set between 0 and 1, the value 0 indicates no similarity and 1 indicates perfect similarity. If the cosine value was set at 0.7, the search would be less sensitive.

Due to the impossibility of reviewing the identified pairs manually, after the linkage was concluded, only pairs that had at least 1 of the variable keys (last name, mother’s name, and date of birth) identical between records were considered true pairs. Those actions increased the specificity of the process. Therefore, the parameters were defined to have a moderate sensitivity and extremely high specificity.

Linkage execution: After defining the parameters and linkage rules, an inner join linkage was performed, where pairs from Sivep-Malaria and Sinan were linked to the SIM data. The “fuzzy_inner_join” function from the “fuzzyjoin” package [23] in R (version 4.1.1) was used for the linkage. Different linkage types can be reviewed in the methodological report by Garcia and colleagues (2022) [2].

## 3. Results

**Figure 1:**
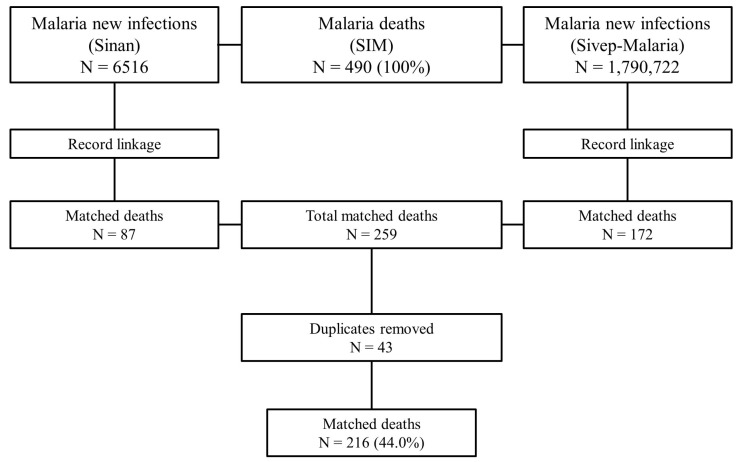
Flowchart of the record linkage process between malaria deaths and cases.

The SIM data totalled 490 deaths between 2011 and 2020, Sivep-Malaria presented 1,790,722 notifications, and Sinan summed 6516 records. After the SIM-Sinan linkage and the exclusion of records considered false positives by the criteria established in the study, 87 pairs were found. The SIM-Sivep linkage resulted in 172 pairs. In total, 259 pairs were found. Of the 259 pairs, 43 duplicates were identified (38 in the SIM-Sivep linkage and 5 in the SIM-Sinan linkage). This left 134 deaths from the SIM-Sivep linkage and 82 from the SIM-Sinan linkage, totalling 216 deaths with recovered information, equivalent to 44.0% of the 490 deaths registered during the study period (Figure 1).

Out of the 216 linked deaths, 148 (68.5%) had a perfect match for the “patient’s name” between the information systems, 117 (54.2%) had a perfect match for the “mother’s name,” and 166 (76.9%) records had a perfect match for the “date of birth.” Regarding the combination of key variables, 65 (30.1%) had a perfect match (identical patient’s name, mother’s name, and date of birth), 85 (39.4%) had a match with 2 identical variables, and 66 (30.5%) had only 1 identical variable (Table 1).

The year with the highest number of matched deaths was 2012 with 32 pairs (48.4% of deaths registered in the year), and the year with the fewest pairs found was 2015, with only 12 pairs (31.5% of deaths in the year). On average, 49 deaths per year were recorded, of which approximately 21 were matched, meaning an average of 44.0% of annual deaths had epidemiological information supplemented from Sivep-Malaria and Sinan data.

The assessment of similarity between the paired data distributions (N = 216) indicated that the proportions of the variables “gender,” “race/colour,” “basic cause of death,” “medical care,” “death investigation,” “year of death occurrence,” and “place of death occurrence” generally had distributions similar to the original database (N = 490) (Table 2). The distribution of the variables “Age Group” and “Education” were the only ones where the distributions were not similar to the original database (Table 2).

Despite the data, in general, having similar distributions, some variables did not have a similar distribution between the linked database and the original databases: there was an expected 14.3% of deaths between 31 and 40 years, but 21.3% were matched (95% CI: 16–27%), indicating more matches between 31 and 40 years than expected. The opposite occurred in the age groups of 71 to 80 years and over 80 years, which had fewer matches than expected (Table 2).

Dissimilar distributions were also found for people with no education; 8.3% were matched (95% CI: 5.2–13.0%) of the deaths, while the expected was 17.6%. For people with more than 11 years of education, 19.4% were matched (95% CI: 15.0–25.0%), and the expected was 11.4%.

Although the chi-square test indicated no difference between the groups, when analysing the variable “Cause of death,” it is observed that there was less matching for deaths with malaria as an associated cause (4.2%; 95% CI: 2.0–8.0%) than expected (9.0%). The same was seen in the variable “State of occurrence,” where fewer deaths were matched (1.4%; 95% CI: 0.3–4.3%) than expected (4.5%) in the state of Acre, and more matched deaths were found in São Paulo (10.6%) than expected (6.7%).

The causes of death described in the SIM showed some consistent data and some inconsistencies when compared with the data recovered from the Sivep-Malaria and Sinan information. Deaths from *Plasmodium falciparum* (N = 54; ICD-10: B50) had 20% (N = 11) inconsistency with epidemiological data, which indicated infections by *Plasmodium vivax* instead of *falciparum*. Deaths from *Plasmodium vivax* (N = 79; B51) had only 5.0% (N = 4) inconsistencies detected. Of the three deaths from *Plasmodium malariae* (B52), two were marked as infections by *Plasmodium falciparum*. The five deaths from “other forms of malaria” (B53) presented positive tests for *Plasmodium falciparum* (N = 1), *Plasmodium vivax* (N = 3), and mixed infections (N = 1). For deaths marked as “unspecified malaria” (N = 66), 39% (N = 26) were infections by *falciparum*, 48% (N = 32) were *vivax* infections, 7.6% (N = 5) were mixed infections, one (1.5%) was caused by *Plasmodium malariae*, one (1.5%) by *Plasmodium ovale*, and one (1.5%) had a result of “non-*falciparum*.” Deaths where malaria was not the underlying cause (N = 9) were seven (78%) *P. vivax* infections, one (11%) *P. falciparum* infection, and one (11%) mixed infection (Table 3).

Regarding the recovery of data for “unknown” or “blank” entries for other variables from the information systems, for the “education” field, 37 (75.5%) records were recovered out of 49 marked as “unknown” or “blank” in the information systems (Table S1); for “race/color,” 8 (72.7%) out of 11 “unknown” or “blank” entries in Sivep-Malaria or Sinan were recovered (Table S2); for the “occupation” field in the SIM and “activity in the last 15 days,” 56 records (60.2%) were recovered from SIM occupation compared to 93 entries marked as “unknown” or “blank” in the “activity in the last 15 days” variable in Sivep-Malaria or Sinan (Table S3).

## 4. Discussion

Approximately 30.6% of information regarding the malaria species causing deaths was not specified in the death certificate. However, record linkage allowed the identification of this information, estimating that almost half of the deaths due to unspecified malaria were caused by *Plasmodium vivax* infections, and nearly 40% were due to *Plasmodium falciparum* infections.

National, state, and municipal malaria death surveillance can use this information to direct disease control actions to prevent deaths. With record linkage, not only can the cause of death be determined, but also the previously nonspecific basic causes can be detailed, enabling more precise information on the locations of deaths (registered in SIM) and the locations of likely infections (registered in Sivep-Malaria and Sinan).

There was a balance between the distributions of cases across variables, indicating that the record linkage technique was able to identify true pair records with similar variable distributions to the original database. That indicates that epidemiological analysis could be carried out using the matched pairs and that those pairs would have a similar distribution to the original dataset.

The combination of information from these HISs allows surveillance services to expand and improve death investigation, enabling the identification of more specific health situations. Initially, the estimate was that about 25.0% of paired deaths were due to *Plasmodium falciparum* and 36.6% to *Plasmodium vivax*. However, with the data recovery, the estimate changes to 37% and 51.4%, respectively. This highlights a weakness in classifying the cause of death in SIM and raises concerns that *Plasmodium falciparum* deaths may be underestimated by almost 50% in Brazil.

The record linkage also allowed the recovery of sociodemographic data related to education level, race/ethnicity, and occupation/economic activities of patients. These pieces of information can be used by epidemiological surveillance services to refine mortality profile analyses and thus direct prevention actions with greater precision.

On the other hand, inconsistencies in the data that were also observed need to be investigated. It is clear that the consistency of information on gender, age group, and race/ethnicity was high (above 92%) because they are basic mandatory filling information on forms. However, information about education showed more than half of the records matched with inconsistencies between SIM data and Sivep-Malaria or Sinan data. Once again, this is information of high importance for supplementing the definition of the mortality profile and health situation analyses to enhance actions aimed at vulnerable groups.

Therefore, it is important for epidemiological surveillance departments to investigate and understand the reasons for these inconsistencies. These inconsistencies may be related to how data are collected during case notification or when filling out the death certificate, and this investigation may reveal other difficulties and limitations in the data collection process.

Furthermore, it is necessary to investigate the unpaired death records in SIM. Due to the sensitivity of this information, this task could not be conducted in this study. It is therefore suggested that the NMCP examine this issue to identify deaths that may not have been reported in the Sivep-Malaria or in the Sinan.

Moreover, as the malaria information systems do not provide specific information on deaths due to the disease, the same applies to the registration of its hospitalisations. Monitoring and investigating factors associated with malaria-related hospitalisations can help prevent such occurrences, thereby potentially reducing deaths. Therefore, it is recommended that this data linkage process also be carried out between the Hospital Information System, Sivep-Malaria, and Sinan.

Additionally, the incorporation of an “outcome” field in the individual notification form of Sivep-Malaria and Sinan should be evaluated in the NMCP. This would enable the tracking of the patient’s progress and the collection of information regarding recovery, disease recurrence, transfers (between health facilities), treatment abandonment, hospitalisations, or death.

Another issue is the lack of a unique identifying variable across different databases. Currently, the HISs are designed with data models that do not have identification documents, such as the CPF, as mandatory record variables in the HIS [2]. The use of record linkage techniques and the undertaking of this work are motivated by the lack of interoperability between the different HISs inside the BMoH. However, the current epidemiological scenarios urge the integration of these different pieces of information, given the difficulty of working with data from various sources [1].

It is important for the IT department responsible for these systems, in conjunction with the health departments, to develop projects and/or updates for the HIS to make this data relatable and make it available to the federated entities. This would enable more qualified analyses of the population’s health status.

Regarding the methods employed in this study, the study achieved a 44.0% data recovery rate out of the total deaths. This indicates low data quality in the systems, particularly with respect to the filling of personal information. Poor data quality has previously been reported as a factor that has a negative influence on probabilistic linkage results [24,25]. This highlights the need to not only train epidemiological surveillance units and technicians but also healthcare professionals responsible for registering death certificates.

Table 1 shows that the use of methods that only allow pairing of records with a perfect match between them results in utilising only 30.1% of the total matched deaths. This highlights that the use of probabilistic methods for data linkage increases the number of linked pairs by at least two times.

The keys used in the linkage process showed a higher frequency of records with “date of birth” correctly filled out between the databases. This may indicate a similar scenario in other databases. Furthermore, among pairs that had only one identical key variable between the systems, “date of birth” allowed the highest number of pairings when the information from the other two key variables was dissimilar (38 pairs; 17.6%) between the systems.

Therefore, it is strongly recommended that the “date of birth” variable be frequently used in probabilistic data linkage processes, as it tends to have fewer filling errors compared to the “patient’s name” and “mother’s name” variables.

Since the linkage rules consider possible spelling errors in filling out key variables for pairing, death records may not have been matched due to an excess of spelling errors in the Sivep-Malaria and Sinan forms. Hence, the low pairing of records for individuals over 71 years of age may be related to lower educational levels. Conversely, among users aged 31 to 40, which had higher pairing rates, the predominant education level is above 8 years of study (Table S4–S6). The same pattern can be observed for deaths recorded with the basic cause “Malaria as an associated cause” (Table S7), as most of these records are of users with “Illiterate” schooling.

The higher or lower pairing than expected for some variables is related to the education level of the deceased, and it is known that elderly people in Brazil have lower levels of education [26]. Therefore, in addition to highlighting that data quality is a critical point to optimise the data linkage process between different HISs, it is also emphasised that epidemiological surveillance technicians should pay closer attention when collecting information from individuals with lower education levels or those in older age groups (elderly).

It is important to note that the definition of rules and parameters for linkage can be adjusted according to the level of data quality to be used. The use of Levenshtein distances [20] and cosine similarity [21] can be calibrated to increase the number of pairs found, but this increase may necessitate a manual review of the pairs to exclude false pairs. Depending on the magnitude of the results, this manual review may become impractical [27]. Also, alternative methods can be investigated, such as the use of deterministic linking methods [2,9] or other packages and functions available for the R software, such as fastlink [28], or even the utilisation of other software like Reclink [29] or Python [30].

In 2018, the General Data Protection Law (LGPD) (Law No. 13,709, of August 14, 2018) [31] was enacted, which emphasises access and the treatment of personal data. This law introduced new rules for accessing information and stricter rules for accessing nominal data for research purposes. LGPD advocates that data provided for research purposes be anonymised whenever possible. For public health research, LGPD allows the sharing of personal data as long as they are exclusively processed within the organisation and strictly for the purpose of conducting studies, these processes can make the research execution process longer than initially planned.

Data linkages have enabled the identification of new information for health surveillance, supporting data quality assessment processes, information supplementation, and the development of public health policies. This information allows for understanding the profile of users in the healthcare field, individual analyses, cohort tracking, and understanding the paths taken by populations through healthcare services.

Finally, this study presents an alternative for the data linkage of the Information Systems in the SUS through free and open statistical and computational techniques. These techniques may allow researchers to analyse data from the same individual across different systems, aggregating a larger number of variables. This enables the identification of morbidity and mortality patterns, which can support the development of public policies that promote and prevent the occurrence of deaths.

Considering that the HISs used in this study are also used by different sectors inside the BMoH [1] it is possible to say that the methods presented are applicable to the surveillance of other death causes such as dengue, HIV/AIDS, tuberculosis, etc. As well, probabilistic linkage techniques to investigate these causes of death have already been reported, but using Reclink software [32,33,34].

The limitations of this study include potential systematic errors arising from the use of secondary databases, such as poor quality of personal data, which may have impacted the total number of pairs identified. Additionally, it is possible that some true pairs were excluded due to the automatic classification of true pairs being the ones with at least one identical key variable among records.

## 5. Conclusions

Probabilistic data linkage techniques enable the relationship of data that lacks a unique identification key. In a scenario where different HISs lack identification variables for users, a probabilistic linkage is a valuable tool for better defining the mortality profile, refining health situation analyses, and increasing the precision of information related to causes of death.

The recovery of epidemiological information on malaria cases for investigating deaths due to the disease is essential to support the National Malaria Control and Prevention Program (NMCP) in monitoring malaria-related deaths and controlling severe malaria cases that may result in death.

It is important that protocols for the acquisition or management of sensitive data are better described at the federal, state, and municipal levels to enable data linkage processes to be carried out by educational and research institutions in addition to the technical units of healthcare services. This collaboration between academia and healthcare services is essential for strengthening public health in the country.

## Figures and Tables

**Table 1:** Quantity of pairings by linkage keys used.

Concordance	Variables Used	Pairs	%
3 variables	Patient’s name	65	30.1%
	Mother’s name		
	Date of birth		
2 variables	Patient’s name	22	10.2%
	Mother’s name		
	Patient’s name	43	19.9%
	Date of birth		
	Mother’s name	20	9.3%
	Date of birth		
1 variable	Patient’s name	18	8.3%
	Mother’s name	10	4.6%
	Date of birth	38	17.6%
Total		216	100.0%

**Table 2:** Profile of malaria mortality and paired deaths between 2011 and 2020, Brazil.

	Total Deaths	%	Paired Deaths	%	95% CI	*p*-Value (Chi-Squared)
	N = 490		N = 216			
Sex						0.8
Male	285	58.2%	128	59.3%	(52–66)	
Female	205	41.8%	88	40.7%	(34–48)	
Age Group						0.014
<1	24	4.9%	9	4.2%	(2.0–8.0)	
1–10	40	8.2%	21	9.7%	(6.3–15)	
11–20	38	7.8%	21	9.7%	(6.3–15)	
21–30	56	11.4%	29	13.4%	(9.3–19)	
31–40	70	14.3%	46	21.3%	(16–27)	
41–50	54	11%	32	14.8%	(10–20)	
51–60	63	12.9%	25	11.6%	(7.8–17)	
61–70	49	10%	14	6.5%	(3.7–11)	
71–80	47	9.6%	9	4.2%	(2.0–8.0)	
>80	48	9.80%	10	4.60%	(2.4–8.6)	
Blank	1	0.20%	-	-	-	
Race/Ethnicity						0.8
White	120	24.50%	53	24.50%	(19–31)	
Black	35	7.10%	14	6.50%	(3.7–11)	
Yellow	2	0.40%	-	-	-	
Brown	258	52.70%	112	51.90%	(45–59)	
Indigenous	65	13.30%	29	13.40%	(9.3–19)	
Blank	10	2%	8	3.70%	(1.7–7.4)	
Years of Education						0.004 *
None	86	17.60%	18	8.30%	(5.2–13)	
1–3 years	70	14.30%	24	11.10%	(7.4–16)	
4–7 years	74	15.10%	32	14.80%	(10–20)	
8–11 years	76	15.50%	44	20.40%	(15–26)	
12 years or more	56	11.40%	42	19.40%	(15–25)	
Ignored	48	9.80%	19	8.80%	(5.5–14)	
Blank	80	16.30%	37	17.10%	(12–23)	
Cause of death (ICD-10)						0.2
B50–*Plasmodium falciparum* (*Pf*)	101	20.6%	54	25%	(19–31)	
B51–*Plasmodium vivax* (*Pv*)	183	37.3%	79	36.6%	(30–43)	
B52–*Plasmodium malariae* (*Pm*)	5	1%	3	1.4%	(0.36–4.3)	
B53–Other Forms	6	1.2%	5	2.3%	(0.86–5.6)	
B54–Not Specified	151	30.8%	66	30.6%	(25–37)	
Malaria–associated cause	44	9%	9	4.2%	(2.0–8.0)	
Received medical care?						0.4
Yes	311	63.5%	150	69.4%	(63–75)	
No	49	10%	17	7.9%	(4.8–13)	
Unknown	21	4.3%	10	4.6%	(2.4–8.6)	
Blank	109	22.2%	39	18.1%	(13–24)	
Was the death investigated?						0.3
Yes	94	19.2%	51	23.6%	(18–30)	
No	236	48.2%	94	43.5%	(37–50)	
Blank	160	32.7%	71	32.9%	(27–40)	
Year of Occurrence						>0.9
2011	72	14.7%	29	13.4%	(9.3–19)	
2012	66	13.5%	32	14.8%	(10–20)	
2013	45	9.2%	19	8.8%	(5.5–14)	
2014	38	7.8%	20	9.3%	(5.9–14)	
2015	38	7.8%	12	5.6%	(3.0–9.7)	
2016	39	8%	19	8.8%	(5.5–14)	
2017	39	8%	15	6.9%	(4.1–11)	
2018	61	12.4%	30	13.9%	(9.7–19)	
2019	39	8%	14	6.5%	(3.7–11)	
2020	53	10.8%	26	12%	(8.2–17)	
State of Occurrence						0.9
Rondônia	37	7.6%	14	6.5%	(3.7–11)	
Acre	22	4.5%	3	1.4%	(0.36–4.3)	
Amazonas	97	19.8%	40	18.5%	(14–24)	
Roraima	51	10.4%	17	7.9%	(4.8–13)	
Pará	73	14.9%	28	13%	(8.9–18)	
Amapá	31	6.3%	18	8.3%	(5.2–13)	
Tocantins	2	0.4%	1	0.5%	(0.02–3.0)	
Maranhão	20	4.1%	4	1.9%	(0.59–5.0)	
Piauí	6	1.2%	4	1.9%	(0.59–5.0)	
Ceará	3	0.6%	1	0.5%	(0.02–3.0)	
Rio Grande do Norte	3	0.6%	1	0.5%	(0.02–3.0)	
Paraíba	2	0.4%	-	-	-	
Pernambuco	7	1.4%	2	0.9%	(0.16–3.7)	
Alagoas	1	0.2%	1	0.5%	(0.02–3.0)	
Sergipe	1	0.2%	1	0.5%	(0.02–3.0)	
Bahia	11	2.2%	6	2.8%	(1.1–6.2)	
Minas Gerais	25	5.1%	13	6%	(3.4–10)	
Espírito Santo	5	1%	3	1.4%	(0.36–4.3)	
Rio de Janeiro	9	1.8%	7	3.2%	(1.4–6.8)	
São Paulo	33	6.7%	23	10.6%	(7.0–16)	
Paraná	5	1%	3	1.4%	(0.36–4.3)	
Santa Catarina	6	1.2%	3	1.4%	(0.36–4.3)	
Rio Grande do Sul	5	1%	3	1.4%	(0.36–4.3)	
Mato Grosso do Sul	2	0.4%	1	0.5%	(0.02–3.0)	
Mato Grosso	16	3.3%	8	3.7%	(1.7–7.4)	
Goiás	10	2.0%	5	2.3%	(0.86–5.6)	
Distrito Federal	7	1.4%	6	2.8%	(1.1–6.2)	

Note: *p-values less than 0.05 are considered statistically significant.

**Table 3:** Basic causes of death (SIM) and laboratory results recovery (Sivep-Malaria and Sinan).

	B50—Pf	B51—Pv	B52—Pm	B53—Other Forms of Malaria	B54—Unspecified Malaria	Malaria—Associated Cause	Total
*Pf*	39 (72.2%)	2 (2.5%)	2 (66.7%)	1 (20.0%)	26 (39.4%)	1 (11.1%)	71 (32.9%)
*Pv*	10 (18.5%)	75 (94.9%)	0 (0.0%)	3 (60.0%)	32 (48.5%)	7 (77.8%)	127 (58.8%)
Mixed	4 (7.4%)	1 (1.3%)	0 (0.0%)	1 (20.0%)	5 (7.6%)	1 (11.1%)	12 (5.6%)
*Pm*	0 (0.0%)	0 (0.0%)	1 (33.3%)	0 (0.0%)	1 (1.5%)	0 (0.0%)	2 (0.9%)
*P. ovale*	1 (1.9%)	0 (0.0%)	0 (0.0%)	0 (0.0%)	1 (1.5%)	0 (0.0%)	2 (0.9%)
Non-*Pf*	0 (0.0%)	1 (1.3%)	0 (0.0%)	0 (0.0%)	1 (1.5%)	0 (0.0%)	2 (0.9%)
Total	54 (25.0%)	79 (36.6%)	3 (1.4%)	5 (2.3%)	66 (30.6%)	9 (4.2%)	216 (100.0%)

## Data Availability

Sensitive information such as names and dates of birth are not available. Other data are available upon reasonable request.

## Supplementary Materials

The following supporting information can be downloaded at: https://www.mdpi.com/article/10.3390/tropicalmed8120519/s1, Supplementary file S1: R script for record linkage; Supplementary file S2: Table S1. Retrieval of “ignored” or “blank” data for the education level variable (in years of study) in SIM, Table S2. Recovery of “ignored” or “blank” data for the race/colour variable in SIM, Table S3. Recovery of “ignored” or “blank” data for the occupation variable in SIM, Table S4. Percentage of matching deaths among people with the underlying cause of death as “Malaria as an associated cause” according to age group in years, Table S5. Percentage of matching deaths of people between 31 and 40 years old according to level of education in years of study, Table S6. Percentage of matching deaths of people over 71 years of age according to level of education in years of study, Table S7. Percentage of matching deaths among people with the underlying cause of death as “Malaria as an associated cause” according to level of education in years of study.
