# Supplementary material for: Record Linkage for Malaria Deaths Data Recovery and Surveillance in Brazil
Source: Trop Med Infect Dis. 2023 Dec 14;8(12):519. doi: 10.3390/tropicalmed8120519 (PMC10748166; doi:10.3390/tropicalmed8120519)
Supplement: Supplementary file 1 [file tropicalmed-08-00519-s001.zip › SUPPLEMENTARY FILE SI.pdf]

Supplementary Material SI

Loading necessary packages

```
library(tidyverse)
library(lubridate)
library(stringr)
library(stringi)
library(fuzzyjoin)
library(stringdist)
library(abjutils)
library(Rcpp)
```

Transforming letters to uppercase, removing invalid connectors or fillers and removing graphic accents

```
SIM$NAME = toupper(SIM$NAME)
SIM$NAME     <- str_replace_all(SIM$NAME,"\,|;|:|-|1|2|3|4|5|6|7|8|9|0| DE | DO |
DAS | DOS |  |IGNORADO|IGNORADA| KG | TEL | OBS | TL | FONE | PESO | DA ",
" " )
SIM$NAME = rm_accent(SIM$NAME)
```

Configuration of binding parameters
Parameter 1

```
dist4 <- function(left, right) {
  stringdist(left, right) < 3
}
```

Parameter 2

```
dist05 <- function(left, right) {
  stringdist(left, right, method="cosine", q=2) < 0.5
}
```

Application of binding parameters to key variables

```
join_SIM_SINAN = fuzzy_inner_join(SIM, SINAN,
                              by = c("NAME", "SURNAME", "NM_MOTHER", "DT_BIRTH"),
                              match_fun = c("NAME" = dist4,
                                            "SURNAME" = dist05,
                                            "NM_MOTHER" = dist05,
                                            "DT_BIRTH" = dist4))
```

```
join_data_SIM_Sivep = fuzzy_inner_join(SIM, Sivep,
                                          by = c("NAME", "SURNAME", "NM_MOTHER",
"DT_BIRTH"),
                                          match_fun = c("NAME" = dist4,
                                                        "SURNAME" = dist05,
                                                        "NM_MOTHER" = dist05,
                                                        "DT_BIRTH" = dist4))
```

Creating parameters to exclude likely false positives

```
#Parameters 1: Pairing of the "patient NAME" key the same between the bases
join_SIM_SINAN$NAME_COMPLETO_IGUAL         <-  ifelse(
join_SIM_SINAN$NAME.1.x         == join_SIM_SINAN$NAME.1.y,  yes = TRUE, no
= FALSE)
```

#Parameters 2: Pairing of the key "BIRTHment date" equal between the bases

```
join_SIM_SINAN$NM_MOTHER_IGUAL         <-  ifelse(
join_SIM_SINAN$NM_MOTHER.x         == join_SIM_SINAN$NM_MOTHER.y,  yes =
TRUE, no = FALSE)
```

#Parameters 3: Pairing of the "mother's NAME" key the same between the bases

```
join_SIM_SINAN$DT_BIRTH_IGUAL         <-  ifelse( join_SIM_SINAN$DT_BIRTH.x
== join_SIM_SINAN$DT_BIRTH.y,  yes = TRUE, no = FALSE)
```

Removing false positives from banks

```
join_SIM_SINAN <- filter(join_SIM_SINAN,
                    join_SIM_SINAN$NAME_COMPLETO_IGUAL == TRUE |
                    join_SIM_SINAN$NM_MOTHER_IGUAL       == TRUE |
                    join_SIM_SINAN$DT_BIRTH_IGUAL      == TRUE )
```
