# Supplementary material for: Record Linkage for Malaria Deaths Data Recovery and Surveillance in Brazil
Source: Trop Med Infect Dis. 2023 Dec 14;8(12):519. doi: 10.3390/tropicalmed8120519 (PMC10748166; doi:10.3390/tropicalmed8120519)
Supplement: Supplementary file 1 [file tropicalmed-08-00519-s001.zip › SUPPLEMENTARY FILE SII.pdf]

SUPPLEMENTARY FILE SII

Supplementary Table S1. Retrieval of “ignored” or “blank” data for the education level variable (in years of study) in SIM.

		Education level in years of study (Sivep-Malaria and Sinan)							
		Illiterate (N = 20)	1 to 3 (N = 26)	4 to 7 (N = 37)	8 to 11 (N = 39)	Over 11 (N = 26)	Ignored (N = 32)	Not applicable (N = 19)	Blank (N = 17)
Education level in years of study (SIM)	Illiterate	8	4	3	0	0	1	1	1
	1 to 3	1	5	7	6	0	2	1	2
	4 to 7	2	6	10	9	0	2	0	3
	8 to 11	1	3	7	13	5	10	0	5
	Over 11	2	0	2	7	20	9	0	2
	Ignored	2	4	7	3	0	3	0	0
	Blank	4	4	1	1	1	5	17	4

Supplementary Table S2. Recovery of “ignored” or “blank” data for the Race/color variable in SIM.

		Race/color (Sivep-Malária e Sinan)					
		White (N = 50)	Black (N = 124)	Yellow (N = 2)	Indigenous (N = 29)	Ignored (N = 8)	Blank (N = 3)
Race/color (SIM)	White	42	6	1	0	3	1
	Black	1	12	0	0	0	1
	Brown	5	103	1	0	3	0
	Indigenous	0	0	0	29	0	0
	Blank	2	3	0	0	2	1

Supplementary Table S3. Recovery of “ignored” or “blank” data for the Occupation variable in SIM.

		Occupation (SIM)	
		Blank (N = 68)	Filled (N = 148)
Occupation (Sivep-Malária and Sinan)	Agriculture	7	22
	Hunting/fishing	2	4
	Construction of roads and dams	0	2
	Domestic	11	20
	Plant exploration	1	0
	Gold Mining	0	8
	Mining	0	3
	Driver	0	3
	Livestock	0	2
	Tourism	1	7
	Traveler	9	21
	Ignored	37	56

Supplementary Table S4. Percentage of matching deaths among people with the underlying cause of death as “Malaria as an associated cause” according to age group in years.

Age range of deaths by "Malaria as an associated cause"	NOT-PAIRED	%	PAIRED	%	Total	%
01 - 10	1	2,86%	1	11,11%	2	4,55%
11 - 20	2	5,71%	-	-	2	4,55%
21 - 30	-	-	2	22,22%	2	4,55%
31 - 40	5	14,29%	2	22,22%	7	15,91%
41 - 50	3	8,57%	1	11,11%	4	9,09%
51 - 60	2	5,71%	1	11,11%	3	6,82%
61 - 70	6	17,14%	1	11,11%	7	15,91%
71 - 80	14	40,00%	-	-	14	31,82%
Over 80	2	5,71%	1	11,11%	3	6,82%

Total	35	100,00%	9	100,00%	44	100,00%

Supplementary Table S5. Percentage of matching deaths of people between 31 and 40 years old according to level of education in years of study.

Education of deaths between 31 and 40 years of age	NOT-PAIRED	%	PAIRED	%	Total	%
Illiterate	3	12,50%	2	4,35%	5	7,14%
1 - 3	2	8,33%	6	13,04%	8	11,43%
4 - 7	3	12,50%	4	8,70%	7	10,00%
8 - 11	7	29,17%	10	21,74%	17	24,29%
Over 11	4	16,67%	15	32,61%	19	27,14%
Ignored	3	12,50%	4	8,70%	7	10,00%
Blank	2	8,33%	5	10,87%	7	10,00%
**Total**	**24**	**100,00%**	**46**	**100,00%**	**70**	**100,00%**

Supplementary Table S6. Percentage of matching deaths of people over 71 years of age according to level of education in years of study.

Education of deaths over 71 years	NOT-PAIRED	%	PAIRED	%	Total	%
Illiterate	38	50,00%	7	36,84%	45	47,37%
1 - 3	15	19,74%	5	26,32%	20	21,05%
4 - 7	9	11,84%	-	-	9	9,47%
8 - 11	2	2,63%	1	5,26%	3	3,16%
Over 11	2	2,63%	2	10,53%	4	4,21%
Ignored	8	10,53%	4	21,05%	12	12,63%
Blank	2	2,63%	-	-	2	2,11%
**Total**	**76**	**100,00%**	**19**	**100,00%**	**95**	**100,00%**

Supplementary Table S7. Percentage of matching deaths among people with the underlying cause of death as “Malaria as an associated cause” according to level of education in years of study.

Education level of deaths by "Malaria as an associated cause"	NOT-PAIRED	%	PAIRED	%	Total geral	%
Illiterate	12	34,29%	1	11,11%	13	29,55%
1 - 3	6	17,14%	1	11,11%	7	15,91%
4 - 7	5	14,29%	2	22,22%	7	15,91%
8 - 11	6	17,14%	1	11,11%	7	15,91%
Over 11	-	-	1	11,11%	1	2,27%
Ignored	4	11,43%	3	33,33%	7	15,91%
Blank	2	5,71%			2	4,55%
**Total**	**35**	**100,00%**	**9**	**100,00%**	**44**	**100,00%**
